# Critical Peripheral Fatigue Thresholds Among Different Force-Velocity Conditions: An Individual-Based Model Approach

**DOI:** 10.3389/fphys.2019.00875

**Published:** 2019-07-16

**Authors:** Baptiste Morel, Thomas Lapole, Cyril Liotard, Christophe Hautier

**Affiliations:** ^1^EA 7424, F-42023, Laboratoire Interuniversitaire de Biologie de la Motricité, Universite de Lyon, Université Jean Monnet Saint-Étienne, Saint-Étienne, France; ^2^Movement-Interactions-Performance, MIP, EA 4334, F-72000, Le Mans Université, Le Mans, France; ^3^EA7424, Laboratoire Interuniversitaire de Biologie de la Motricité, Université Claude Bernard Lyon 1, Villeurbanne, France

**Keywords:** evoked torque, voluntary activation, group III/IV muscle afferents, exercise, performance

## Abstract

During high intensity exercise, metabosensitive muscle afferents are thought to inhibit the motor drive command to restrict the level of peripheral fatigue to an individual’s critical threshold. No evidence exists of an individual relationship between peripheral fatigue and the decrease in voluntary activation reached after prolonged all-out exercise. Moreover, there is no explanation for the previously reported large decrease in voluntary activation despite low metabolic stress during high force contractions. Thirteen active men completed two maximal intensity isokinetic knee extension tests (160 contractions) under conditions of low force – high velocity and high force – low velocity. Neuromuscular testing including maximal torque, evoked torque and voluntary activation, was done every 20 contractions. The exponential modeling of these variables over time allowed us to predict the stable state (asymptote) and the rate of decrease (curvature constant). For both high and low force contractions the evoked torque and voluntary activation asymptotes were negatively correlated (*R*^2^ = 0.49 and *R*^2^ = 0.46, respectively). The evoked torque asymptotes of the high and low force conditions were positively correlated (*R*^2^ = 0.49). For the high force contractions, the evoked torque and voluntary activation curvature constant were negatively correlated (*R*^2^ = 0.43). These results support the idea that a restrained central motor drive keeps peripheral fatigue under this threshold. Furthermore, an individual would show similar fatigue sensibility regardless of the force generated. These data also suggest that the decrease in voluntary activation might not have been triggered by peripheral perturbations during the first high force contractions.

## Introduction

Muscle fatigue can be defined as an exercise-induced reduction in maximal voluntary muscle force (Gandevia, 2001). This phenomenon may arise from many sites along the neuromuscular system, i.e., from the initiation of the motor drive to the cross bridge cycle (Bigland-Ritchie and Woods, 1984). Most studies distinguish central fatigue, a decrease in neural activation of the musclesdue to numerous spinal and supraspinal factors (Gandevia, 2001), from peripheral fatigue, an attenuated contractile response to neural input induced by biochemical changes at the myocyte level (Allen et al., 2008; Debold, 2012; Westerblad, 2016). However, as the last decade has provided abundant evidence of interactions between central and peripheral fatigue (Taylor et al., 2016; Blain and Hureau, 2017), this simplistic model no longer appears pertinent.

Specific neural pathways, including feed forward and feedback processes, seem to be involved in the connections between the brain and the muscles during fatigue (Hureau et al., 2016b). The feed forward process refers to corollary discharge (Wolpert et al., 1995), a neural signal generated in the brain’s motor centers, which activates sensory areas within the cortex, influencing perception of the effort and participating in central fatigue (Gallagher et al., 2001; Liu et al., 2005). Feedback processes involve various mechano- and metabo-sensitive muscle afferents. It has been found that group Ia, Ib, and II spindle afferents are able to modulate their discharge rate during sustained or repeated contractions and thus reduced excitatory or increase inhibitory inputs to the motor neurons (Gandevia, 2001). However, their role in muscle fatigue appears to be minimal (McNeil et al., 2011). Conversely, group III and IV afferents have clearly been shown to significantly influence the development of fatigue (Amann and Dempsey, 2016; Blain et al., 2016; Matsuura, 2016). These muscle afferents are free nerve endings activated by contraction-induced mechanical and chemical stimuli (Rowell and O’Leary, 1990; Kaufman et al., 2002). Group III afferents are more responsive to mechanical stimuli such as muscle contraction or stretch than group IV afferents and may provide information to the central nervous system about the force of muscular contractions (Kaufman et al., 2002). In contrast, group IV afferents are the most sensitive to metabolic by-products and respond quickly to perturbations in muscle homeostasis (Kaufman et al., 2002).

Several studies have demonstrated the existence of a “*critical threshold of peripheral fatigue*.” This hypothetical construct is based on the idea that a negative feedback loop involving group III/IV muscle afferents operates to protect the muscle from severe threats to homeostasis during whole-body exercise (Amann and Dempsey, 2008; Amann et al., 2009; Blain et al., 2016). Although Babault et al. (2006) and Morel et al. (2015a) did not design their studies to address questions concerning the “critical threshold” concept, they interestingly compared maximal intensity repeated knee extensions that differed in their contraction mode and/or force-velocity contraction characteristic. Despite similar intensity (i.e., maximal), the mechanical characteristic of the contraction (i.e., force and velocity) produce different neuro-physiological responses. For instance, while higher force contractions (i.e., during low velocity contractions) can produce intramuscular blood flow occlusion (Zwarts and Arendt-Nielsen, 1988), the higher velocity contractions (i.e., with lower force production) are associated with a greater metabolic activity (Dorel et al., 2003). As a result, it was reported that “higher force – lower velocity” contractions rapidly induce a significant decrease in voluntary activation which precedes (Babault et al., 2006) and/or limits (Morel et al., 2015a) the decrease in evoked torque. As, the “*critical threshold of peripheral fatigue*” is not applicable to all exercise modalities (Hureau et al., 2016b). It might be that the development of central fatigue during repeated “*high force – low velocity*” contractions is not solely related to increased intramuscular metabolic perturbations. The “sensory tolerance limit,” another more global concept, may be interesting to consider here. This theoretical concept purports that exercise intensity is regulated by a negative feedback loop that is the sum of all feedback (i.e., not only feedback from the metabolic activation of the active group III/IV muscle afferents) and feed forward mechanisms to ensure that voluntary activity remains tolerable (Hureau et al., 2016b). Better understanding the force decrease mechanisms resulting from high-force contractions would allow us to specifically optimize training. This may be critical for athletes needing to develop high forces (Morel et al., 2015b) but also deconditioned people which performed daily tasks at near maximal force capacity (Hortobágyi et al., 2003).

Previous studies of this threshold concept have all used sustained tasks such as cycling (Dekerle et al., 2008; Hureau et al., 2016a), knee extensions (Burnley, 2009) or handgrips (Kellawan and Tschakovsky, 2014). During such exercises, fatigue was seen to be characterized by a hyperbolic decay of power or torque with time (Poole et al., 2016). This model is mathematically defined by an asymptote generally interpreted as the highest intensity (i.e., force, velocity or power depending on the exercise modality) at which a physiological steady-state can be achieved, e.g., for oxygen uptake (Poole et al., 2008), blood flow (Copp et al., 2010), intramuscular concentration of inorganic phosphate, phosphocreatine and hydrogen ions (Jones et al., 2008). Unfortunately, to our knowledge, no study has yet modeled the corresponding decreases in voluntary activation and evoked torque with decreasing force/power over time which could help us understand the interactions between the central and peripheral fatigues during maximal-intensity exercise. Furthermore, it has recently been suggested that the consistency of the loss of force threshold observed across studies (in agreement with the threshold concept) may be an “artifact” of aggregated data (Neyroud et al., 2016). These authors consider that critical thresholds (global or peripheral) would be relevant only at the subject level making the analysis of individual data, and not just aggregated data, necessary.

The aim of the present study was therefore to analyze, on an individual basis, the time course of the decreases in voluntary activation and evoked torque during repeated maximal contractions. Repeated maximal intensity isokinetic knee extensions were performed following two force-velocity characteristics of contraction: high *versus* low velocities allowing the production of low *versus* high forces, respectively. We hypothesized that (i) the time course for the decrease in voluntary activation observed for the low force contractions would be positively correlated to the decrease in evoked torque thus confirming the concept of a critical threshold for peripheral fatigue in this condition, (ii) the higher forces produced in the low velocity condition would additionally induce central fatigue but dissociated from the time course for the peripheral fatigue.

## Materials and Methods

### Subjects

Thirteen active men volunteered to participate in this study (Mean ± SD; age: 22.1 ± 3.8 year; mass: 74.3 ± 8.2 kg; height: 1.81 ± 0.06 m; self-reported training volume: 7.0 ± 3.3 h.wk^-1^). All participants were involved in collective sports with mixed aerobic and resistance training. Written informed consent was obtained from the participants, and the study was conducted according to the declaration of Helsinki. Approval for the project was obtained from the local ethics committee for human research (CPP Sud Est I, France).

### Experimental Design

**FIGURE 1:**
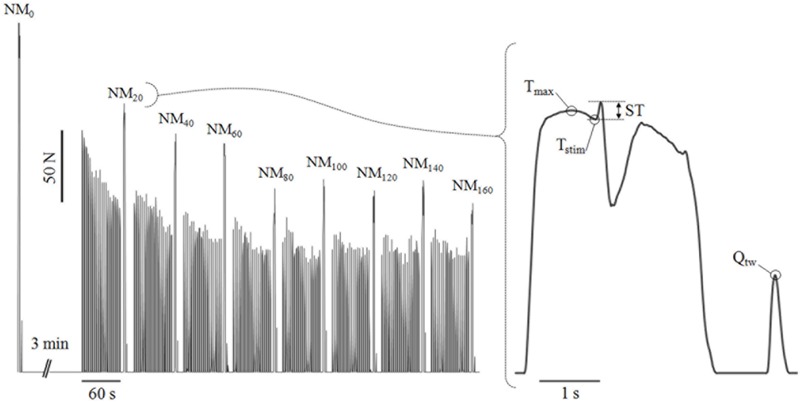
Typical torque traces obtained during the fatigue procedure **(left panel)** and a neuromuscular measurement **(right panel)**. An isometric neuromuscular measurement was conducted after every twenty 1–s contractions performed either at 30°.s^-1^ or 90°.s^-1^. NM_x_, neuromuscular measurements after *x* seconds of accumulated contraction time; T_stim_, torque level at the point of the stimulation; T_max_, maximal torque produced before the stimulation; ST, superimposed twitch, i.e., difference between the maximal torque attained after the stimulation and Ts_tim_; Q_tw_, resting potentiated twitch, i.e., maximal torque response following a high frequency doublet stimulation on the relaxed potentiated muscle.

The subjects first participated in a session to familiarize them with the testing procedures. Two force conditions (low force: LF; high force: HF) were then tested during two randomly ordered experimental sessions, separated by a week but performed at the same time of day. Neuromuscular measurements and the fatigue testing procedure were done on an isokinetic dynamometer (Contrex MJ; Dübendorf, Switzerland; sensitivity 0.5 N.m). The participants were seated with their hips at 100° (180° is full extension) and secured with straps across the chest, hips and the thigh of the working leg (dominant side, right for all participants) to avoid lateral and frontal displacement. They were also asked to cross their arms over their chests during the voluntary contractions to limit the involvement of core muscles. The leg was fixed to the fulcrum with a 40 mm strap placed 2 cm proximal to the medial malleolus. After preparation and determination of the optimal intensity for femoral nerve stimulation (see below), subjects performed a standardized warm-up (8 × 240°.s^-1^; 6 × 180°.s^-1^; 4 × 90°.s^-1^; 2 × 30°.s^-1^; 2 × 0°.s^-1^; with 1 min rests between sets; intensity increased progressively to 90% of maximal perceived effort at the end of the warm up). Neuromuscular measurements of the knee extensors were then made 3 min before and then every 20 contractions during the fatigue testing (without any rest) and immediately after the last (160th) contraction (Figure 1).

### Fatigue Testing Procedure

Two contraction velocities were used (30°.s^-1^: HF; 90°.s^-1^: LF) for the isokinetic knee extensions at maximal voluntary intensity. The range of motion was adapted to each contraction velocity to standardize the contraction time (1 s) and provide a similar “central point” of 120° (180° is full extension) for the resting length of the quadriceps muscle (i.e., 30°.s^-1^: 105°–135°; 90°.s^-1^: 75°–165°). Fatigue testing consisted of eight sets of 20 repetitions of a maximal 1-s isokinetic knee extension and a 2-s passive flexion. Neuromuscular measurements were made in the 15-s period separating each set. The fatiguing exercise lasted 10 min in total. As previously demonstrated in the literature, such a duration is sufficient to reach the asymptote (i.e., stable state) of the torque–time relationship (Burnley, 2009; Kellawan and Tschakovsky, 2014). During all voluntary contractions, the subjects were instructed to “push as hard and as fast as possible” and were provided with real-time visual feedback allowing them to monitor the torque produced during each maximal knee extension. Participants were given strong verbal encouragement throughout the exercise.

### Electrical Stimulation of the Femoral Nerve

The femoral nerve was stimulated percutaneously using a self-adhesive cathode (10-mm diameter, Ag-AgCl, Contrôle Graphique Medical, Brie-Comte-Robert, France) stuck manually over the femoral triangle. The anode, a 10 × 5 cm self-adhesive stimulation electrode (Medicompex SA, Ecublens, Switzerland), was placed over the gluteal fold. The best position for the anode was that which maximized the twitch response and minimized the M-wave of the biceps femoris. Squarewave stimuli of 200 μs duration with a maximal voltage of 400 V were delivered with a constant current (Digitimer DS7AH, Hertfordshire, United Kingdom). The supramaximal stimulation intensity was determined for each experimental session by progressively increasing the current until there was no further increase in the evoked isometric twitch response. The last intensity obtained was then increased by 20% to ensure a supramaximal stimulus (optimal intensity: 158 ± 45 mA).

### Measurements and Data Analysis

The isometric torque was measured with the isokinetic dynamometer during all the neuromuscular test procedures and stored at a sampling rate of 2 kHz on a computer using an analog-to-digital converter (Power Laboratory/16SP; AD Instruments, Australia). Subjects were requested to perform one isometric maximal voluntary contraction (IMVC; duration: about 4 s) with the knee at 120°. For the baseline measurements, if the plateau did not reach at least 95% of the control IMVC performed at the end of the warm up, the procedure was stopped and a new one started after a 1-min rest. During the IMVC, a high-frequency doublet stimulus (100 Hz) was delivered when maximal torque was reached (visually controlled) while the subjects were asked to continue producing maximal effort for about 1 s after the stimulation. Another doublet stimulation (100 Hz) was performed on the relaxed muscle in a potentiated state 2-s later (Figure 1). The whole procedure lasted less than 15 s. The IMVC torque plateau was calculated as the mean torque over a 500-ms period prior to the superimposed twitch. The voluntary activation (VA) was calculated using equation 1. The evoked torque (Q_tw_) was calculated as the maximal torque response following a high frequency doublet stimulation of the relaxed, potentiated muscle. IMVC, VA, and Q_tw_ measured during and after testing were then expressed as a percentage of the pre-fatigue values.

**Equation 1.** Voluntary activation calculation (Strojnik and Komi, 1998). VA, Voluntary activation; T_stim_, torque level at the point of the stimulation; T_max_, maximal torque produced before the stimulation; ST, Superimposed Twitch, i.e., the difference between the maximal torque attained after stimulation and Ts_tim_; Q_tw_, resting potentiated twitch, i.e., the maximal torque response following a high frequency doublet stimulation on the relaxed, potentiated muscle.

$$\text{VA}=[1-[\text{ST}\times ({\text{T}}_{\text{stim}}/{\text{T}}_{\max})]/{\text{Q}}_{\text{tw}}]\times 100$$

### Statistical Analysis

All data were analyzed with Statistica 8.0 Software (StatSoft Inc.,^®^ Tulsa, OK, United States) and expressed as means ± standard deviation. Non-linear regression techniques were used to fit the kinetics of the dependent variables, i.e., IMVC, Q_tw_ and VA for the independent variable: contraction time [equation 2 (Hendrix et al., 2009)] and for each subject. An iterative process was used to minimize the sum of the squared error between the fitted function and the observed values.

**Equation 2.** Equation used for the non-linear regression. Dependent Variable (DV, i.e., IMVC, Q_tw_, or VA) and A (asymptote) are expressed as a percentage of the value measured during the pre-fatigue neuromuscular test; t, accumulated contraction time in seconds; τ, curvature constant in seconds.

$$\text{DV}(\text{Time})=\text{A}+(100-\text{A})\times {\text{e}}^{\left(-\text{t}/\tau \right)}$$

In order to compare alterations in voluntary activation and evoked torque for a given IMVC decrease, we also expressed both VA and Q_tw_ as a function of torque. To do this, from equation 2, time can be expressed as a function of IMVC which gives,

(3) $$t=-\tau_{\text{IMVC}}\times \ln\left(\frac{{\text{IMVC-A}}_{\text{IMVC}}}{100-{\text{A}}_{\text{IMVC}}}\right)$$

Substituting equation 3 in equation 2 gives,

(4) $$\text{DV}={\text{A}}_{\text{DV}}+(100-{\text{A}}_{\text{DV}})\times {\left(\frac{{\text{IMVC-A}}_{\text{IMVC}}}{100-{\text{A}}_{\text{IMVC}}}\right)}^{\frac{\tau_{\text{IMVC}}}{\tau_{\text{DV}}}}$$

with the dependent variable (DV) being VA or Q_tw_. Equation 4 was then used to estimate on an individual basis the VA and Q_tw_ for 10, 20, and 30% IMVC.

With the assumption of normality and homogeneity of variance confirmed, paired *t*-tests with Bonferroni correction were employed to test the force condition (HF vs. LF) on the asymptote and curvature constant for IMVC, Q_tw_ and VA Data as well as VA and Q_tw_ estimated for 10%, 20% and 30% IMVC decrease (Equation 4). Within each force condition, a simple linear regression was used to verify the influence of A_V A_ on A_Qtw_ and τ_V A_ on τ_Qtw_. A simple linear regression was also used to verify the influence of A_Qtw-HF_ on A_Qtw-LF_. For the regression analysis, the coefficient of determination (R^2^) was used to calculate the correlations between the two scores. For all tests, the alpha level for statistical significance was set at *p* < 0.05.

## Results

### Individual Models

All the individual exponential regressions were statistically significant for IMVC, VA, and Q_tw_ for both HF and LF conditions (all *p* < 0.05). Each parameters of the exponential modeling are provided for each individual in Table 1.

**Table 1:** Individual exponential models of fatigue for high force (HF) and low force (LF) conditions.

	HF									LF								
	IMVC			VA			Q_tw_			IMVC			VA			Q_tw_		
	A (%)	τ (s)	R^2^	A (%)	τ (s)	R^2^	A (%)	τ (s)	R^2^	A (%)	τ (s)	R^2^	A (%)	τ (s)	R^2^	A (%)	τ (s)	R^2^
#01	64	34	0.81	69	95	0.81	71	31	0.94	47	42	0.98	68	107	0.94	67	79	0.95
#02	65	52	0.96	83	18	0.96	76	76	0.92	53	75	0.96	88	161	0.96	59	49	0.98
#03	66	32	0.92	85	8	0.83	77	56	0.90	45	65	0.98	79	106	0.76	56	25	0.98
#04	73	19	0.72	83	14	0.76	63	75	0.89	56	19	0.98	81	131	0.61	58	91	0.98
#05	70	17	0.69	56	11	0.98	84	75	0.96	32	117	0.88	67	110	0.99	71	68	0.91
#06	63	25	0.94	90	71	0.92	74	48	0.96	63	23	0.98	80	42	0.92	70	23	0.98
#07	74	12	0.90	83	10	0.81	75	66	0.97	55	37	0.88	57	133	0.90	73	28	0.96
#08	59	33	0.98	74	51	0.92	77	59	0.94	51	24	0.94	60	41	0.79	70	8	0.88
#09	73	61	0.90	91	32	0.94	73	51	0.90	76	45	0.92	82	33	0.83	70	51	0.86
#10	51	33	0.94	97	80	0.98	55	16	0.90	41	90	0.96	59	136	0.63	58	75	0.98
#11	70	17	0.81	78	60	0.89	77	30	0.99	65	62	0.90	81	62	0.96	61	29	0.98
#12	55	24	0.85	84	70	0.81	73	17	0.96	46	29	0.98	61	27	0.83	79	14	0.88
#13	48	42	0.96	97	35	0.99	50	42	0.98	56	27	0.96	97	86	0.88	41	28	0.94

**Mean**	**64**	**31**	**0.88**	**82**	**43**	**0.89**	**71**	**49**	**0.94**	**53^∗^**	**50^∗^**	**0.95**	**74^∗^**	**90^∗^**	**0.85**	**63^∗^**	**44**	**0.94**
**SD**	**9**	**14**	**0.09**	**11**	**30**	**0.08**	**10**	**21**	**0.03**	**11**	**30**	**0.04**	**13**	**45**	**0.12**	**11**	**27**	**0.04**

*Asymptote (A), curvature constant (τ), and coefficient of determination (R^*2*^) are provided for each modeling of isometric maximal voluntary contractions (IMVC), voluntary activation (VA), and evoked torque (Q_*tw*_) parameters. Mean and standard deviation (SD) are provided for each variable. ^∗^Statistically different from the same variable in the HF condition.*

### Force Condition Effect Based on Pooled Data

**FIGURE 2:**
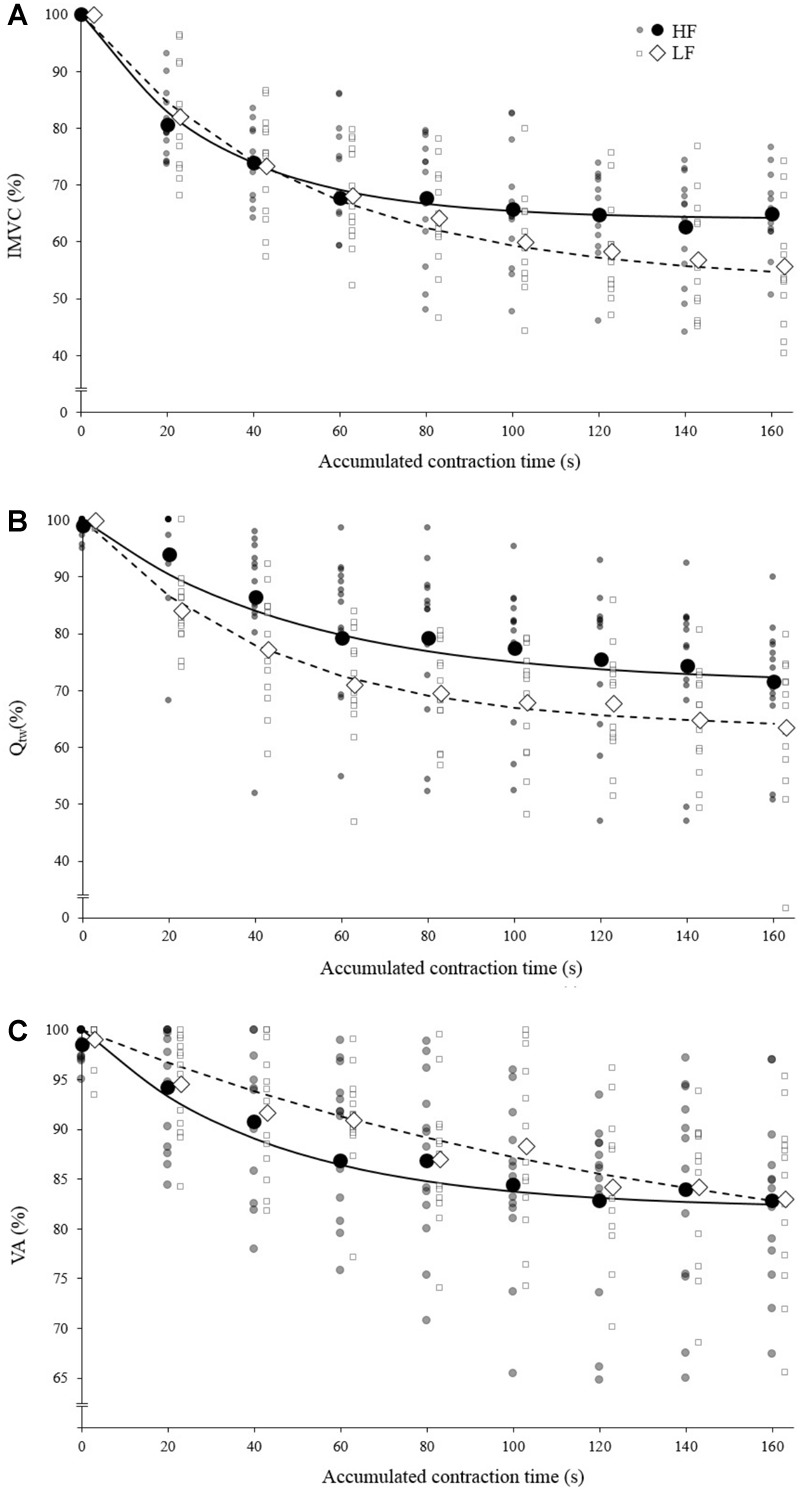
Mean fatigue kinetics for isokinetic knee extensions performed with high force (HF; black) and low force (LF; white). Individual data are represented by the small markers. **(A)** Torque (Isometric Maximal Voluntary Contraction) – time relationship. **(B)** Evoked torque (Q_tw_) – time relationship. **(C)** Voluntary activation (VA) – time relationship; IMVC, Q_tw_ and VA are expressed as a percentage of the maximal value. The solid/dotted line corresponds to exponential modeling of the relationship for HF and LF, respectively. Markers representing HF and LF conditions are slightly shifted for a visualization purpose.

Both the first contraction and the asymptote absolute peak torque were statistically higher at 30°.s^-1^ compared to 90°.s^-1^ (1st contraction: 234 ± 36 N for HF vs. 184 ± 29 N for LF, *p* < 0.001; Asymptote: 155 ± 17 N for HF vs. 112 ± 21 for LF, *p* < 0.001). The absolute IMVC baseline values were not statistically different between LF and HF for IMVC (LF: 275 ± 64 N.m; HF: 285 ± 62 N.m), VA (LF: 94.7 ± 4.7%; HF: 96.9 ± 4.4%) and Q_tw_ (LF: 96 ± 18 N.m; HF: 96 ± 19 N.m). For IMVC, the asymptote was lower (*p* = 0.005) and the curvature constant greater (*p* = 0.044) for LF compared to HF (Figure 2A and Table 1). For Q_tw_, the asymptote was lower for LF compared to HF (*p* = 0.025) but the curvature constant was not significantly different (*p* = 0.544; Figure 2B and Table 1). For VA, the asymptote was lower (*p* = 0.001) and the curvature constant greater (*p* = 0.004) for LF compared to HF (Figure 2C and Table 1).

**FIGURE 3:**
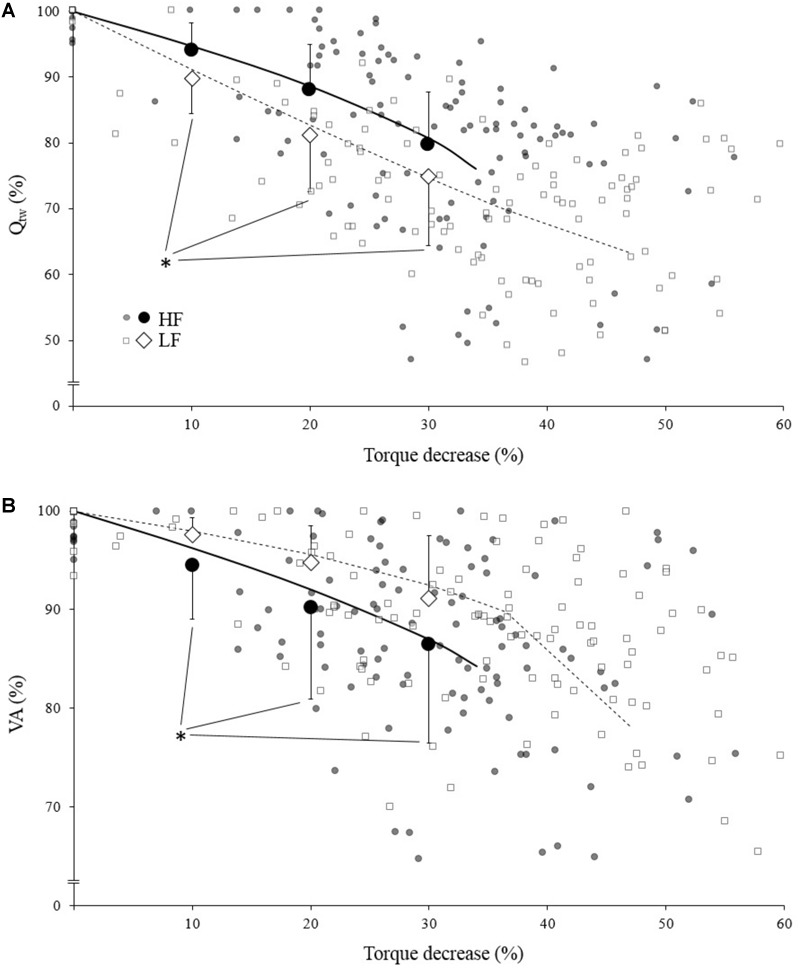
Mean evoked torque (Q_tw_; panel **A**) and voluntary activation (VA; panel **B**)expressed as a function of torque (IMVC) decrease for high force (HF; black) and low force (LF; white) condition. Each neuromuscular testing are represented by the small markers. The big markers represent the mean (pooled data) for VA/Q_tw_ decrease at 10, 20, and 30% IMVC decrease estimated from individual models and Equation 4. All data are expressed as a percentage of the maximal value obtained during the pre-fatigue neuromuscular testing. The solid/dotted line corresponds to mean exponential modeling of the relationship for HF and LF, respectively. ^∗^Statistical difference between HF and LF.

When compared to a similar IMVC decrease, Q_tw_ decrease was significantly more important for LF compare to HF (Figure 3A; *p* = 0.006, <0.001, and <0.001 for 10, 20, and 30%, respectively). Conversely VA decrease was significantly more important for HF compare to LF (Figure 3B; *p* = 0.036, <0.001, and <0.001 for 10, 20, and 30%, respectively).

### Evoked Force and Voluntary Activation Correlations Based on Individual Data

**FIGURE 4:**
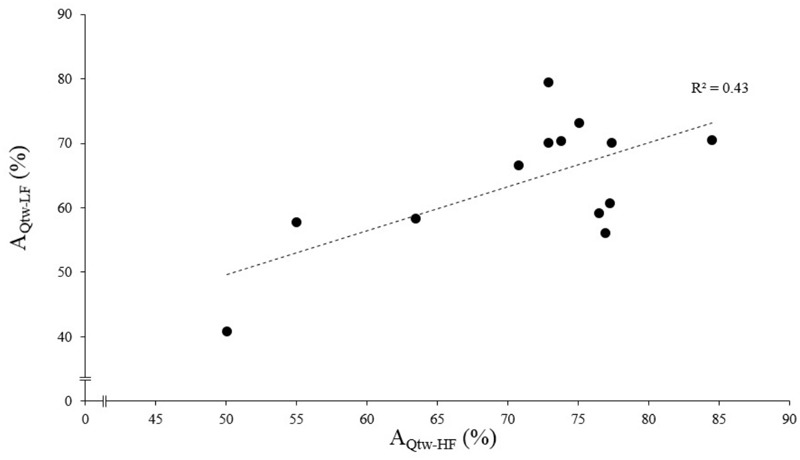
Evoked torque asymptote correlation for high force (HF) and low force (LF) condition. Each dot represent an individual. The evoked torque asymptote for HF (A_Qtw-HF_) and LF (A_Qtw-LF_) are expressed as a percentage of the maximal value obtained during the pre-fatigue neuromuscular testing. The correlation was statistically significant, *p* < 0.001.

**FIGURE 5:**
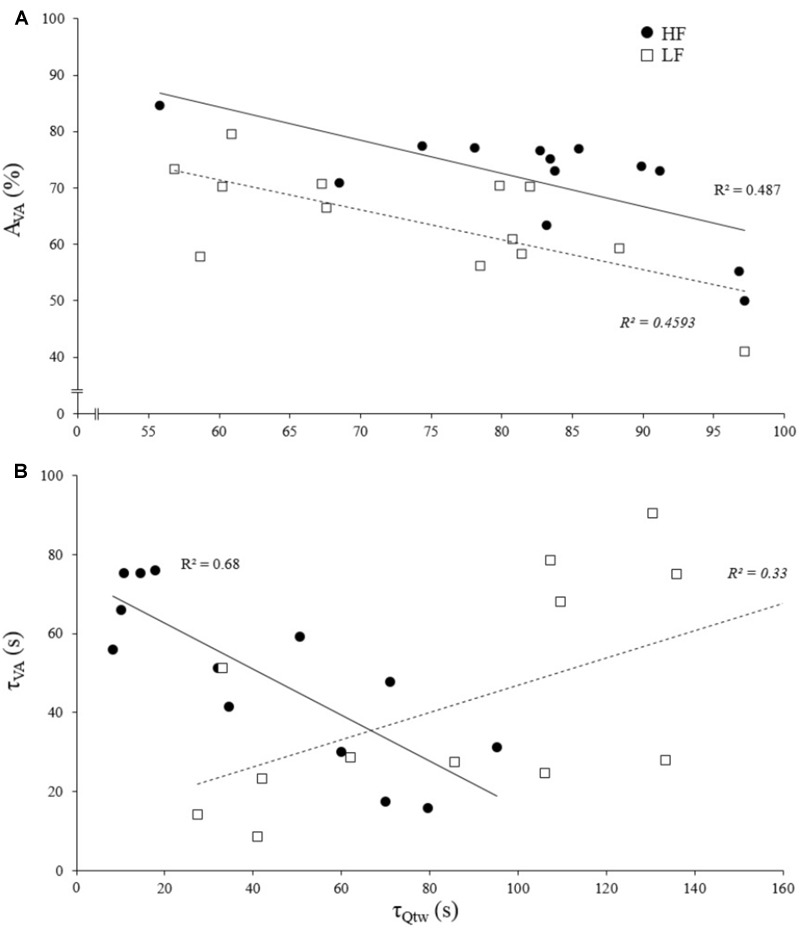
Correlation of evoked torque and voluntary activation for asymptotes and curvature constant of the exponential fatigue kinetics model. Each dot represent an individual. **(A)** Evoked torque (Q_tw_) and voluntary activation (VA) asymptote correlations for knee extensions performed with high force (HF) and low force (LF). **(B)** Evoked torque (Q_tw_) and voluntary activation (VA) curvature constant (τ) correlations for knee extensions performed with high force (HF) and low force (LF). All correlations are statistically significant, *p* < 0.05.

The asymptotes of HF and LF were statistically correlated for Q_tw_ (*p* = 0.014, *R*^2^ = 0.43; Figure 4) but not for VA and IMVC (respectively, *R*^2^ = 0.11 and 0.10; *p* = 0.268 and 0.293). The asymptotes of VA and Q_tw_ were negatively correlated for HF (*p* = 0.008; *R*^2^ = 0.49) and LF (*p* = 0.011; *R*^2^ = 0.46; Figure 5A). The curvature constant of VA and Q_tw_ were negatively correlated for HF (*p* < 0.001; *R*^2^ = 0.68) and positively correlated for LF (*p* = 0.039; *R*^2^ = 0.33) (Figure 5B).

## Discussion

The purpose of the present study was to analyze the individual time course of central and peripheral contributions to force loss during prolonged exercise performed at maximal voluntary intensity with low vs. high force using high vs. low velocity isokinetic knee extensions, respectively. The major results were that (i) the decrease in muscle contractility is limited to different critical thresholds in low vs. high force conditions, in the context of data modeled on an individual basis; (ii) the significant correlation between the stable states for voluntary activation and evoked torque modeled in each condition reinforces the idea that central inhibitions keep the peripheral fatigue under a critical threshold; (iii) the inverse correlation between the rate of voluntary activation and the decrease in evoked torque during high force contractions suggests that, in this condition, central inhibition was not triggered by peripheral perturbations at the start of exercise.

The analysis of the torque–time relationship evidenced that the torque losses observed at the end of the exercise (i.e., asymptote) were greater for the lowest force condition (Figure 2A) which is in line with previous studies (Mathiassen, 1989; Callahan et al., 2009; Morel et al., 2015a). Furthermore, the curvature constant was almost twice lower for HF. Mathematically, 95% of the torque loss is achieved when 3 τ elapsed, independently of the asymptote value, i.e., 90-s of accumulated contraction time for HF ad 150-s of accumulated contraction time for LF (Figure 2A). In other words, fatigue develops faster during low force contractions. The analysis of evoked torque and voluntary activation time courses will allow a better understanding of the observed differences.

At the end of the exercise the decrease in evoked torque was significantly greater for the lowest force contractions (Figure 2B) which is consistent with the results previously reported by Babault et al. (2006) and Morel et al. (2015a). Moreover, even when expressed for a given IMVC decrease to account for a possible dose response, the evoked torque decrease was greater for the lowest force condition (Figure 3A). A loss of muscle contractility is commonly associated with an accumulation of intramuscular metabolites (e.g., inorganic phosphate and adenosine diphosphate) (Debold, 2012; Westerblad, 2016). Since the mean power produced in the high force condition was lower (i.e., ≈ 60 W vs. ≈ 120 W at the end of the exercise) it can be hypothesized that the metabolic power and thus the intramuscular metabolic perturbations were lower in this condition (Barclay, 1996; Dorel et al., 2003). Since an individual’s “*critical peripheral fatigue threshold*” is considered to limit exercise capacity (Amann and Dempsey, 2016; Blain et al., 2016; Poole et al., 2016) it is interesting to observe that in the present study the decrease in evoked torque was confined to a critical threshold which differs between low and high force conditions. The fact that the subjects present largely different thresholds (from 20 to 60% of evoked torque loss) may be explained by endogenous reference signals (Matsuura, 2016). Indeed, some authors claim that numerous factors such as training history, muscle substrate reserves, muscle metabolic rates, prior experience or mental fatigue alter the interpretation of group III/IV muscle afferent feedback and may generate different individual responses (Lambert et al., 2005; Marcora, 2009; Marcora et al., 2009). Interestingly, the peripheral fatigue thresholds of LF and HF were positively correlated among our subjects (Figure 4). Consequently, an individual would be more or less sensitive to peripheral perturbations but would remain so regardless of the exercise. This may explain the mathematical relationship between the peripheral thresholds for HF and LF. An analysis of the decrease in voluntary activation with repeated contractions may provide insight into the inhibitory mechanisms and the contractile losses.

The exponential modeling indicated that the voluntary activation asymptote was significantly lower for LF (Figure 2C). This remains exact when the voluntary activation decrease is normalized to the IMVC decrease (Figure 3B). However one should keep in mind that the curvature constant was really high in this condition and thus the fatigue testing procedure was not long enough to allow the subject to reach the stable state. Previous studies reported a greater decrease in voluntary activation with isometric or low velocity contractions compared to higher contraction velocities (Babault et al., 2006; Morel et al., 2015a) or no differences between conditions (Callahan et al., 2009). Considering the entire voluntary activation-time relationship these discrepancies previously observed can be explained by the time of the neuromuscular testing. For example, in the present study, the voluntary activation can be different after 60 s but not after 120 s when comparing the two force conditions (Figure 2C). This demonstrates the interest of recording and modeling the development of peripheral and central fatigue over time. Considering the greater loss of contractility seen during LF (see above), the greater decrease of voluntary activation estimated in this condition may be explained by peripheral perturbations, possibly through activation of the III-IV afferent inhibitory pathways. Originally, the curvature constant of the model (τ) indicated a faster decrease in voluntary activation for HF despite lower peripheral fatigue. Thus, in this condition it appears unlikely that central fatigue has been mediated by peripheral perturbations. However, these results come from mean analysis of pooled data while Neyroud et al. (2016) recently raised the point that critical thresholds would be relevant only at the individual level. Hence, we propose below to consider individual rather than averaged data.

The Figure 5A shows that asymptotes for the decreases in voluntary activation and evoked torque were negatively correlated. This supports the concept of a “*critical peripheral fatigue threshold*.” Whatever the exercise modality tested in the present study, the development of “peripheral fatigue” was kept under this critical threshold. It is worth noting that very important inter-individual differences occurred despite a rather homogeneous population (i.e., all were active young men). Indeed, some participants were able to maintain almost 100% of the initial voluntary activation level but demonstrated a 60% evoked torque decrease, whereas others experienced a 50% decrease in voluntary activation but only a 20% evoked torque decrease. It appears that each participant was uniquely sensitive to the peripheral perturbations that regulate the descending motor drive. All this reinforces the idea discussed above that each individual is more or less sensitive to intramuscular metabolic perturbations possibly due to factors such as training status (Zghal et al., 2015) or psychological aspects (e.g., motivation, anxiety, mental stress) (Lambert et al., 2005). Another interest of this time-course model is to quantify the rate at which evoked torque or voluntary activation decreases. Indeed, the curvature constants (τ) for voluntary activation and evoked torque over time were positively correlated for LF (Figure 5B). It could be hypothesized that, in this condition, the rate at which metabolites accumulated influenced the rate at which voluntary activation decreased via metabo-activation of group III-IV afferents as previously suggested (Blain et al., 2016; Matsuura, 2016). Interestingly, this was apparently not the case during HF since a negative correlation was observed (Figure 5B). For each individual, the faster the drop in voluntary activation, the slower the decrease in muscle contractility. The mechanisms involved in this interrelation between central and peripheral mechanisms cannot be identified in the present study and one should keep in mind that correlation does not imply causation. Nevertheless, in this condition, central fatigue might not have been triggered by peripheral homeostasis perturbations at the very beginning of the exercise. Other feedback such as mechano-activation of group III afferents might have been involved in response to the high level of force developed (Kaufman et al., 2002) (peak torque for HF was twice higher than for LF). Hence, central inhibition mighty have preceded and limited the intramuscular metabolic perturbations during the beginning of the contractions.

The aim of this study design was to fix the contraction mode, contraction time, number of contractions, duty cycle and intensity all known to influence the development of fatigue (Enoka, 1995) in order to analyze the sole effect of the force-velocity characteristic of a muscle contraction. Unfortunately, the range of motion had to differ in order to control the others parameters; angular positions influence muscle activation and so force production (Pincivero et al., 2004), and may have influenced the development of fatigue. The greater range of motion during LF has probably contributed to the lower force in this condition since force capacity and voluntary activation decreased when the angle moved away from the optimal angle (i.e., 120°) (Noorkõiv et al., 2014). Nevertheless, the order of magnitude of the differences between the range of motion used for HF and LF is less than 20% for the torque and less than 10% for the voluntary activation (Noorkõiv et al., 2014). This is thus relatively small compared to the factor two between the peak forces for HL vs. LF and may not have mainly accounted for the observed differences.

In summary, this study has demonstrated that peripheral fatigue is maintained under a critical threshold which is specific to the force conditions, in the context of data modeled on an individual basis. The individual thresholds for the decreases in voluntary activation and evoked torque modeled in each condition are negatively correlated which support the idea that a restrained central motor drive keeps peripheral fatigue under this threshold. Furthermore, although the average thresholds for peripheral fatigue differed between low *versus* high force contractions, the present results show that an individual would show similar fatigue sensibility regardless of the force generated. Finally, the negative correlation between the rate of the decrease in voluntary activation and evoked torque during the high force contractions suggests that, in this condition, central inhibition was not triggered by peripheral perturbations at the very beginning of the exercise. Other feedback (e.g., mechanical) or feed forward processes require further study in order to better apprehend the development of neuromuscular fatigue especially during high force contractions.

## Ethics Statement

This study was carried out in accordance with the recommendations of the local ethics committee (CPP Sud Est I) with written informed consent from all subjects. All subjects gave written informed consent in accordance with the Declaration of Helsinki.

## Author Contributions

BM, TL, and CH conceived and designed the experiments, and wrote the manuscript. BM, TL, and CL performed the experiments. BM, TL, CL, and CH analyzed the data and contributed materials and analysis tools.

## Conflict of Interest Statement

The authors declare that the research was conducted in the absence of any commercial or financial relationships that could be construed as a potential conflict of interest.
